# Supplementary material for: Visual analytics framework for survival analysis and biomarker discovery from gene expression data
Source: PLoS One. 2026 Mar 20;21(3):e0325399. doi: 10.1371/journal.pone.0325399 (PMC13004513; doi:10.1371/journal.pone.0325399)
Supplement: Attachment — Submitted filename: response-to-reviews.pdf [file pone.0325399.s001.pdf]

RESPONSE TO THE REVIEWERS

Dear Reviewers,

we thank for the comprehensive feedback that helped us to improve the manuscript, sufficient time to implement the changes, and the permission to submit a revised version. Below, we provide the detailed response to reviewers.

Kind regards,

Jaka Kokošar, Cagatay Turkay, Luka Ausec, Miha Štajdohar and Blaž Zupan

Ljubljana, November 21, 2025

Reviewer #1

This article presents a visual analysis methodology designed to simplify survival analysis in biomedicine. It proposes a framework of reusable visualization and modeling components, akin to "LEGO bricks," to aid in biomarker discovery. This approach addresses the limitations of existing methods that often require programming skills or are inflexible, thus hindering accessibility for biomedical researchers. Its goal is to provide a user-friendly, open-source tool (implemented as an Orange Data Mining add-on) that facilitates interactive data exploration and hypothesis-driven research. It is relevant for the journal. However, I have some suggestion for improvement shown below.

> 1. The article provides a valuable tool for survival data analysis, but could be enhanced by explicitly addressing scalability for large and complex datasets.

We thank the reviewer for highlighting this important point. We have clarified scalability in the revised manuscript by explicitly noting in the Discussion section that the framework's performance scales with the underlying Python ecosystem (NumPy, pandas, scikit-learn) and therefore supports datasets of similar size and complexity. We have also updated the METABRIC dataset description to better contextualize dataset size in our case studies.

> 2. Future improvements could focus on advanced interactive visualization techniques to uncover intricate relationships within high-dimensional data, potentially incorporating machine learning-driven insights for predictive modeling and automated pattern detection.

We thank the reviewer for this suggestion. We have addressed it by expanding the Discussion section with a new use case that shows how our survival analysis components integrate with other machine-learning, bioinformatics and data visualization approaches. We also added a general statement clarifying that our framework integrates well with essentially any machine-learning method available, and that achieving this level of integration is there by design.

> 3. Expanding the tool's interoperability with other popular biomedical data platforms would broaden its adoption.

Agreed. This is an important point we have addressed with a new use case in the Discussion section. Please see the answer above (r1q2).

> 4. A detailed discussion of how the tool supports causal inference in survival analysis, beyond mere correlation, would significantly enhance its analytical capabilities.

This comment is inline with previous comments on integrating other functionalities in our framework. We agree, and have addressed it with a new use case and discussion about it in the Discussion, also with a note that "This flexibility also means that users can incorporate analytical techniques beyond simple correlation-based exploration. For example, ssGSEA we have included in our use case is a representative of such feature-group modeling approaches."

> 5. Please improve the quality of the figures.

All figures included in the work are screenshots submitted in losesslless format (PNG) and should reproduce well in the journal. We carefully checked them again and will take care that readbility in not lost due to scaling in the offprints.

Reviewer #2

> 6. The paper describes a visual analytics tool for survival analysis. It presents substantial work and is potentially interesting for a broad readership. The paper is well-written and easy to follow. My major concern is the novelty of the work.

To address this comment, we added a paragraph in Introduction that explicitly lists the main contributions of the paper (the task-oriented decomposition of survival analysis, the design of reusable visual analytics components, and the use-case based demonstration of utility).

> 7. As contribution, the authors mention support for cohort definition and cohort discovery. Highly relevant papers in this area (also with medical applications), however, are not even cited:
>
> - Supporting iterative cohort construction with visual temporal queries. J Krause, A Perer, H Stavropoulos. IEEE Transactions on Visualization and Computer Graphics 22 (1), 91-100.
> - Interactive visual patient cohort analysis. Z Zhang, D Gotz, A Perer. Proc. of IEEE VisWeek Workshop on Visual Analytics in Health Care.
> - Somarakis, A., Ijsselsteijn, M. E., Luk, S. J., Kenkhuis, B., de Miranda, N. F., Lelieveldt, B. P., & Höllt, T. (2020). *Visual cohort comparison for spatial single-cell omics-data*. IEEE Transactions on Visualization and Computer Graphics, 27(2), 733–743.
> - Preim, B., Klemm, P., Hauser, H., Hegenscheid, K., Oeltze, S., Toennies, K., & Völzke, H. (2016). *Visual analytics of image-centric cohort studies in epidemiology.* In *Visualization in Medicine and Life Sciences III* (pp. 221–248). Springer.
>
> Thus, the contributions should be either better justified or revised.

We thank the reviewer for pointing us to these important works on interactive cohort-based analysis. We agree they make significant contributions to cohort construction and comparison for specific data modalities. Our goal, however, is not to introduce a new cohort-discovery algorithm or domain-specific cohort interface, but to provide a compact set of reusable, workflow-oriented visual components that integrate established survival-analysis methods within an interpreted visual-programming environment. While the framework supports cohort-driven analyses, developing specialized cohort-definition techniques for EHRs, spatial omics, or imaging data is outside the scope of this work. Accordingly, we have revised the contribution statement in the Introduction to place less emphasis on cohort discovery.

> 8. "many clinicians and life science researchers" - I would remove "clinicians". Without very few exceptions, clinicians, i.e., the persons treating patients under severe time pressure, is not the target group for this research.

We thank the reviewer for this helpful suggestion. Agreed — frontline clinicians are not the primary audience. We have revised the manuscript by replacing "clinicians" with "clinical researchers" to reflect the intended user group more accurately.

> 9. I also wonder whether the title of the paper is ideal. Of course, the method is not restricted to cancer, not even to medicine, but cancer patients is by far the most important application, also discussed in the paper. Reading the abstract, I thought, what this might be good for and clearly for cancer patients. But cancer is neither part of the abstract, nor the title. The authors should at least consider to change this

Agreed; we would like to thank the reviewer for this suggestion. We have revised the title and to narrow the scope ("Visual analytics framework for survival analysis and biomarker discovery from gene expression data") and have updated the abstract by adding a sentence highlighting cancer as the primary application area.

> 10. I would be more convinced of the paper if the tool indeed helped to gain new insights. I like the idea of insight-based evaluations (Chris North) for visual analytics applications. I would not say that such an evaluation is mandatory for publishing this work, but to better understand examples of true insights would be helpful. I understand that the author did a lot to apply their tool to real world data, also to confirm existing hypotheses - that is also useful, but I miss the aha-moment: Here is something that is very hardly possible with any other tool; the uniqueness. Applying visual analytics to e.g., gene-related data - that is really not new.

We thank the reviewer for this suggestion. To address it, we added a new use-case in the Discussion that (1) applies previously published data in a new, exploratory way rather than focusing on replication, and (2) demonstrates (potential) a-ha moments through unexpected gene-set rankings, marker prioritization, and the identification of an interpretable gene-set–based marker. Our new use case should illustrate how the proposed framework supports rapid prototyping of analytically complex workflows that can expose useful insights. We appreciate the reference to insight-based evaluation frameworks by Chris North, however, as the reviewer notes, such comprehensive user studies are beyond the scope of this work, especially given that they would need to evaluate the entire analytical environment rather than a single visualization technique as proposed by North and colleagues (Saraiya et al., IEEE TVCG, 2005).

> 11. The terms "clinical event of interest" and "clinical outcomes" have strongly overlapping, if not identical meanings. I recommend to either clarify what discriminates both terms or use just one of them.

Agreed. We have consolidated the terminology and now consistently use the term *clinical outcomes* throughout the manuscript.

> 12. The term "interpreted workflow" is interesting. I suggest to emphasize it and elaborate it.

Agreed. We revised the Related Work section (subsection on Orange data mining framework) and explicitly define interpreted workflows, provided a reference where this name was introduced, and explain why they are essential for enabling real-time interaction in our visual analytics setting.

> 13. Figure 3: I would use the term brush-and-link at least in "()" in the caption.

We revised the caption of Figure 3 to explicitly include the term "brush-and-link" when describing the interaction between the Distribution and Kaplan–Meier Plot widgets.

14. References: A couple of conference references may be shortened, e.g., 18, 21, 23. They contain the year twice and IEEE twice.

We revised the references to remove the duplication and to align them with the journal's reference style.
